# Supplementary material for: Pathophysiological Significance of Neutrophilic Transfer RNA-Derived Small RNAs in Asymptomatic Moyamoya Disease
Source: Cells. 2021 May 1;10(5):1086. doi: 10.3390/cells10051086 (PMC8147334; doi:10.3390/cells10051086)

2D Structure

```
83                     8mer-1a        97
5 ' - c c A G A G T T C A C A G G a - 3 ' utr
          | : | |   | | | | | | |
3 ' - g u U U U C - A G U G U C C g - 5 ' tRF
16 15   14 13         7 6 5 4 3 2
3' Pairing                 Seed
```

Local AU

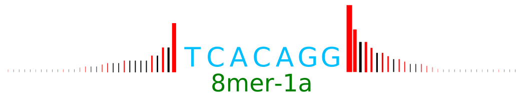

Position

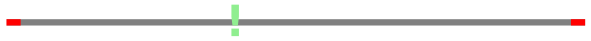

Supplement: Supplementary file 1 [file cells-10-01086-s001.zip › cells-1149161 3rd supplementary materials/Data Supplements/Data Supplement V.zip!/Data supplement 6-mRNAs targets of tsRNAs/tsRNA-Arg-CCT-008/Diagrams/tRF-Arg-CCT-008_vs_ENST00000404031(AC131097.2).pdf]
